# Crop/Plant Modeling Supports Plant Breeding: II. Guidance of Functional Plant Phenotyping for Trait Discovery

**DOI:** 10.34133/plantphenomics.0091

**Published:** 2023-09-28

**Authors:** Pengpeng Zhang, Jingyao Huang, Yuntao Ma, Xiujuan Wang, Mengzhen Kang, Youhong Song

**Affiliations:** ^1^ School of Agronomy, Anhui Agricultural University, Hefei, Anhui Province 230036, China.; ^2^ College of Land Science and Technology, China Agricultural University, Beijing 100094, China.; ^3^ The State Key Laboratory for Management and Control of Complex Systems, Institute of Automation, Chinese Academy of Sciences, Beijing 100190, China.; ^4^ Centre for Crop Science, Queensland Alliance for Agriculture and Food Innovation, The University of Queensland, Brisbane, QLD 4350, Australia.

## Abstract

Observable morphological traits are widely employed in plant phenotyping for breeding use, which are often the external phenotypes driven by a chain of functional actions in plants. Identifying and phenotyping inherently functional traits for crop improvement toward high yields or adaptation to harsh environments remains a major challenge. Prediction of whole-plant performance in functional–structural plant models (FSPMs) is driven by plant growth algorithms based on organ scale wrapped up with micro-environments. In particular, the models are flexible for scaling down or up through specific functions at the organ nexus, allowing the prediction of crop system behaviors from the genome to the field. As such, by virtue of FSPMs, model parameters that determine organogenesis, development, biomass production, allocation, and morphogenesis from a molecular to the whole plant level can be profiled systematically and made readily available for phenotyping. FSPMs can provide rich functional traits representing biological regulatory mechanisms at various scales in a dynamic system, e.g., Rubisco carboxylation rate, mesophyll conductance, specific leaf nitrogen, radiation use efficiency, and source–sink ratio apart from morphological traits. High-throughput phenotyping such traits is also discussed, which provides an unprecedented opportunity to evolve FSPMs. This will accelerate the co-evolution of FSPMs and plant phenomics, and thus improving breeding efficiency. To expand the great promise of FSPMs in crop science, FSPMs still need more effort in multiscale, mechanistic, reproductive organ, and root system modeling. In summary, this study demonstrates that FSPMs are invaluable tools in guiding functional trait phenotyping at various scales and can thus provide abundant functional targets for phenotyping toward crop improvement.

## Introduction

To meet the demands for greater food supply, changes in diet, and sustainable biofuel consumption, global crop production needs to be doubled by 2050 [1–3]. This requires advancing crop breeding and associating agronomical practice in delivering increased yield under abiotic stress worsened by climate change [4–7]. Over the past decades, the efforts on advanced biotechnologies in promoting breeding technology to molecular design breeding (version 4.0) will greatly improve precise breeding and shorten the breeding cycle [5,8–11]. However, breeders still face challenges in identifying traits at various levels that can be used in guiding breeding directions by employing a large amount of genomic information [4,11–13]. Therefore, the identification of desired phenotypic traits allowing high-throughput phenotyping in breeding programs is urgently needed.

Plant phenomics is the multidisciplinary study of accurate acquisition and analysis of multidimensional phenotypes on plant growth, development, yield, and composition in a high-throughput fashion [14,15]. With the rapid development of machine vision, artificial intelligence, big data computing, and analysis, plant phenomics has made substantial progress in phenotyping a wide range of traits [16–18], especially morphological traits under different scenarios for breeding use (Table 1). Phenotyping morphological traits has been proven essential and successful in crop improvement programs since three-dimensional (3D) architecture determines the capacity of plant to capture light or nutrients, such as dwarfing wheat and rice [19] and upright maize and sorghum [20,21]. However, the internal complex physiological and biochemical traits at the various scales that determine external morphological traits are often overlooked [22]. Functional traits link the genotypes and external phenotypes, by which the genetic information is translated into external phenotype, generate knowledge about how plants operate at the physiological level, and work synergistically with forward genetics [22–25]. That is, functional phenotyping aids in narrowing down the specific trait to be phenotyped, thus efficiently selecting promising breeding candidates and avoiding wasted resources in recording large amounts of data [26–28]. Furthermore, the relationship between morphological features and physiological functions is also neglected during the phenotypic analysis processes [24,29]. Recent research has demonstrated the potential for improved genetic gains by linking morphological and functional traits rather than using morphological traits alone in situations where genotype × environment interactions are significant in breeding programs [24,28]. Unfortunately, there are only a few examples of functional trait analysis actually being applied to obtain superior crop lines [28], mainly due to technical limitations in phenotyping functional traits at the population scale that lag behind breeding techniques [30,31]. As a consequence, more emphasis on plant phenomics should urgently be placed on phenotyping functional traits to narrow the genotype-to-phenotype knowledge gap in future breeding programs.

**Table 1.** Commonly used sensors for noninvasive acquisition of plant phenotypes.

Sensors	Property description	Morphological traits	Functional traits	Pros and cons	References
RGB cameras	Create color images with red, green, and blue in the spectral region of 400 to 700 nm	Growth dynamic, phenology, green-area, leaf rolling/angle/wilting, photosynthetically active radiation area, canopy structure/coverage/height, stay-green/senescence, lodging, ear/plant density, grain number and size, biomass, yield, diseases, and pests	Chlorophyll/nitrogen content, nitrogen use efficiency, nitrogen nutrition index, seedling vigor	Low cost and easy, spatial resolution, signal-to-noise ratio, throughput, environmental adaptation, repeatability	[130–139]
				Specific wavelength missing, limited physiological process, susceptible to illumination	
Multispectral cameras	Simultaneously acquire spatial images in many spectrally contiguous bands	Growth dynamic, phenology, green-area, leaf rolling/wilting, photosynthetically active radiation area, canopy coverage/height/structure, stay-green/senescence, lodging, plant density, biomass, yield, diseases and pests	Chlorophyll/nitrogen/water content, nitrogen use efficiency, nitrogen nutrition index, water use efficiency, stomatal conductance, grain quality	Fast and easy	[137–140]
				Susceptible to weather and plant architecture, limited spectral bands, frequently calibrated	
Hyperspectral cameras	Simultaneously acquire spatial images in many spectrally contiguous bands	Growth dynamic, phenology, green-area, leaf rolling/wilting, photosynthetically active radiation area, canopy coverage/structure, stay-green/senescence, lodging, biomass, yield, diseases and pests	Chlorophyll content, nitrogen content, nitrogen use efficiency, nitrogen nutrition index, water content, water use efficiency, net photosynthesis, grain quality	High prediction accuracy, measurement at wide range; identify limited disease	[141–145]
				Susceptible to weather and plant architecture, data analysis difficult, expensive, and frequently calibrated	
Thermal infrared cameras	Measure the surface temperature by detecting infrared radiation, which is emitted by objects at a temperature higher than absolute zero	Green-area, leaf rolling/wilting, canopy coverage, lodging, yield diseases, and pests	Canopy temperature, stomatal conductance, water content, water use efficiency	Fast and easy	[12,40,137,140]
				Susceptible to weather and plant architecture, need soil background correction, difficult to detect very small temperature differences, the high-resolution sensor is expensive	
Chlorophyll fluorescence	Map the emitted chlorophyll fluorescence signal (650 to 800 nm) to the sample space on a pixel basis	Projected leaf area, lodging, biomass, yield, diseases, and pests	Nonphotochemical quenching, photosynthetic electron transport rate, intrinsic light-harvesting efficiency, light use efficiency, nitrogen use efficiency, nitrogen nutrition index, grain quality	Sensitively to stress	[2,146,147]
				Strict protocol, difficult to image deep complex canopies, reproducibility, data analysis difficult	
Synthetic aperture radar	Detect radar echoes to produce high-resolution 3D images	Plant identification, phenology, normalized difference vegetation index, planting area, lodging, yield		Fast, large-scale, not influenced by clouds and haze	[148–150]
				Expensive, data analysis is difficult, specific wavelength missing, radiometric calibration, atmospheric and geometric correction	
Ground penetrating radar	Map sub-surface structures by measuring reflection, refraction, and scattering of pulses of high-frequency radio waves	Root bulk, root biomass		Higher precision, resolution, automatic, high-throughput	[151,152]
				Cannot detect fine roots, limited ability to detect genotypic differences	
LiDAR	Measure the distance from objects to sensors using a single-beam laser operated in the NIR or SWIR regions and can form a point cloud	Growth dynamic, phenology, green-area, leaf rolling/angle, photosynthetically active radiation area, canopy coverage/height/structure, lodging, ear density, grain number and size, biomass, yield		High-accuracy, real-time, all directions, low noise	[118,140,153,154]
				Expensive, susceptible to weather, data processing, limited physiological processes	
X-ray computed tomography	Detect the differences in energy absorption before and after scanning from different angles	Internal and external 3D structure, tiller morphology, panicle growth	Water content, flow speed, grain quality	High spatial resolution, non-invasive and non-destructive 3D images, repeatability, signal-to-noise ratio	[155,156]
				Cost and scanning time, low-throughput, poor environmental adaptation	
Magnetic resonance imagers	Detect energy release when protons realign with the magnetic field	Internal 3D structure, root structure, root diseases	Water content, metabolic study	High spatial resolution, simple sample preparation, fast, measurement of the whole plant and internal cell	[18,67,157]
				Limited resolution in giant cells, susceptible to soil elements, radiation effects	
Positron emission tomography	Radiotracer mapping and co-registration with positron emission signals		Water transport, flow velocity	Quantitative and spatial information	[158–160]
				Limited spatial resolution, cost time	
Raman imaging	Detect variations in molecular vibrations or rotations caused by excitation of inelastic scattering of sample photons	Internal structure	Compositional fingerprints	High resolution, reasonable signal-to-noise ratio, repeatable	[18,161,162]
				Small sample volume, burning the tissue with laser illumination, high autofluorescence	

Constrained by phenotyping techniques, noninvasive and high-throughput analyses of underlying functional traits are far behind the morphological features since the latter is easier to be caught by imaging systems [23,26,27,29]. Thus, there is a great demand for tools to identify fundamentally functional traits. Crop/plant models may be effective tools in identifying robust functional traits under different environments for breeding use [6,32]. Such models allow the dissection of complex phenotypes into the most likely set of mechanisms, thus aiding in identifying key functional traits for crop breeding [33], and offer another approach to quantifying the potential benefit of trait modification in target scenarios [34]. For example, the daily biomass production resulting from environmental conditions and internal functional traits of the given genotype can be dissected into daily incident solar radiation, multiplied by the fraction of interception (depending on leaf area) and energy conversion efficiency (depending on leaf photosynthetic capacity), and reduced energy loss through respiration (mitochondrial respiration) [35]. As a result, crop/plant modeling is attracting increasing attention from scientists in different disciplines, regarded as a key component in agricultural systems to address the challenges of food security, sustainability, and climate change [6]. In this study, we examined the technical challenges facing plant phenomics and then highlight the great potential of crop/plant modeling in phenotyping plant functional features. Therefore, we demonstrate the role of functional–structural plant models (FSPMs) in guiding functional phenotyping for breeding use by exemplifying the premium properties in robust functional–structural feedback, flexible integration across multiple scales, and provision of rich sources of functional targets.

## Plant Phenomics Appeals for Tools for Functional Trait Identification

Functional phenotyping requires profiling plant functions at various scales [15,22,36], which fills important knowledge gaps between genomic information and the external phenotypes for complex traits [24,25,27,37]. Application of phenotyping functional traits in breeding can enhance the precision and genetic gains for complex trait [29], and avoid attempts to quantify emergent properties expressed at the whole plant/population level [28]. For instance, Coupel-Ledru et al. [38] used a functional trait phenotyping approach, demonstrating that reducing nocturnal transpiration is a win–win strategy for improving grape transpiration efficiency and ensuring growth, and can be a new potential target for grape breeding. In a similar way, Pang et al. [39] found a positive correlation between leaf transpiration and phosphorus uptake based on data from 266 chickpea genotypes and revealed a nonintuitive relationship between mass flow and phosphorus uptake.

Unfortunately, there are limited studies on applying functional phenotyping in breeding programs [28] since functional phenotyping is relatively sophisticated, complex, and costly. The inability to screen numerous genotypes for specific functional traits has been a critical limitation in phenotyping for breeding. The major reason is due to lack of functional targets. Although experimental methods can test the expression of a trait by modifying it in assessing its effect on crop yield, they may be expensive, impractical, and even impossible to realize in traditional multisite, multiseason cultivation trials [28]. Performance at the whole plant level results from accumulated responses at the cellular, tissue, and organ levels to various abiotic factors [14,23,40]. This requires an effective tool in assisting to systematically dissect plant growth and development into secondary functional traits [15,33], such as early vigor, Rubisco carboxylation rate, electron transport rate, specific leaf nitrogen, assimilate allocation, etc. Confronting with potentially complex reactions, mechanistic understanding is the key in identifying target functional traits [41]. FSPMs have been proven to be a promising tool to realize such aims [32,42–44] due to the ability of predicting the phenotypic performance cumulatively from the interaction between organ morphogenesis and physiological functioning under various environments [45,46].

## Features of FSPMs

In the last century, researchers have been working to develop models of plant development, growth, and function [47–50], such as agricultural production systems simulator (APSIM) [34], rice crop model (ORYZA) [51], and genotype-by-environment interaction on crop growth simulator (GECROS) [37]. Although these models perform well in predicting crop yield, there are still some limitations because a set of assumptions and some key processes in crop models are simplified, making it impossible to extrapolate to new scenarios and identify trait variations in detail [52]. Such issues can be exemplified like (a) ignoring the effect of initial seed reserves [53], planting density [54], and morphological variability within canopy [55]; (b) inaccurate prediction of green leaf area index (LAI) [37]; (c) difficult to integrate aging dynamics [56]; and (d) unable to display in 3D [57].

**Fig. 1.**
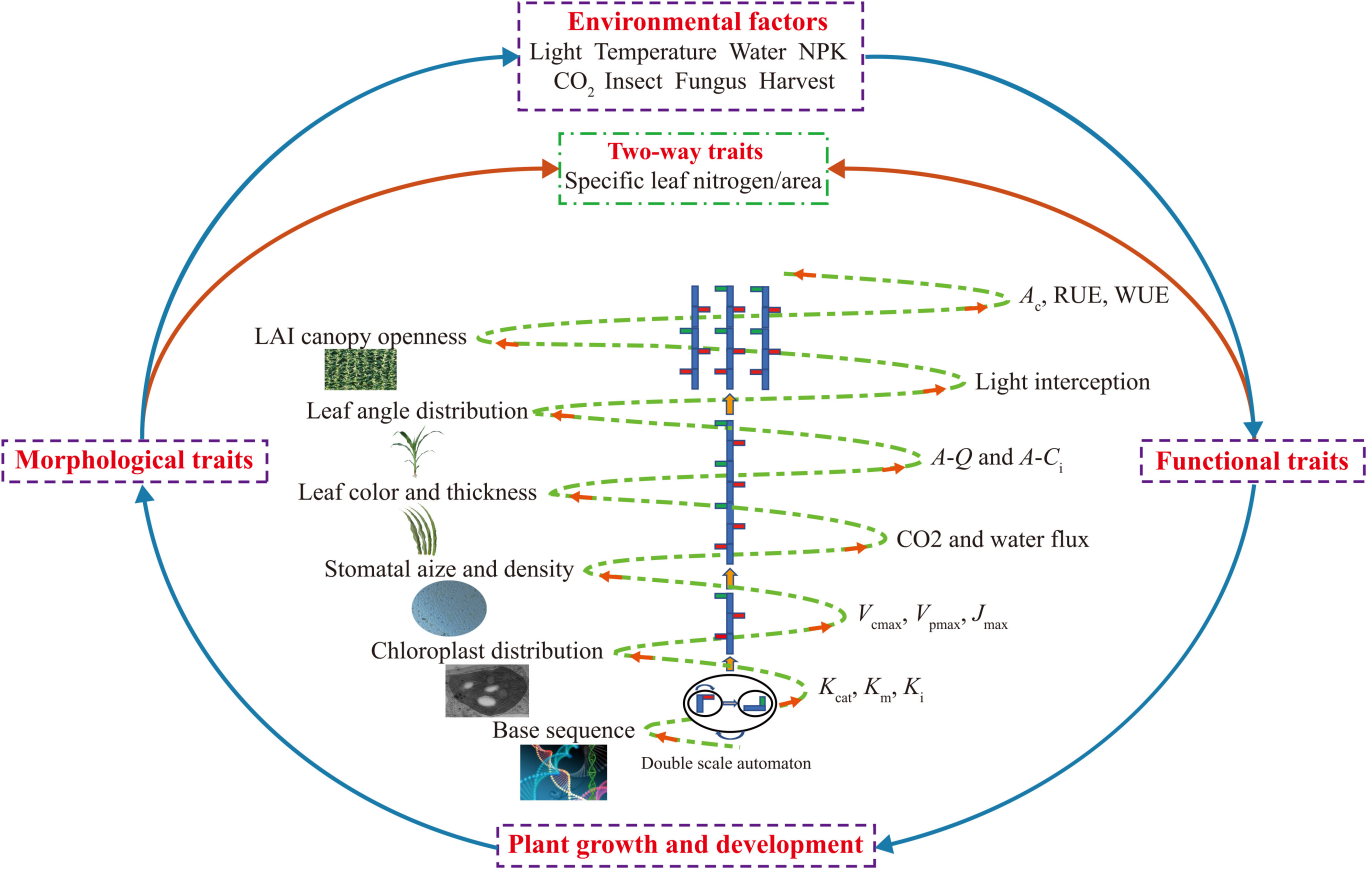
FSPMs capture a feedback loop between structure, function, and environment, which can be used to scale from gene to population (center of the figure: temporal and spatial scales spiral). Outputs from models describing processes at the lower scales can be used as inputs to models describing processes at higher scales. All this information is updated at each time step of the model (*K*_cat_: catalytic number, *K*_m_: Michaelis–Menten kinetics, *K*_i_: inhibition constant, *V*_cmax_: maximum Rubisco activity, *V*_pmax_: maximum PEPC activity, *J*_max_: maximal linear electron transport rate, *A–Q*: photosynthetic light response, *A–C*_i_: photosynthetic intercellular CO_2_ response, *A*_c_: canopy photosynthesis, RUE: radiation use efficiency, WUE: water use efficiency).

In the 1990s, FSPMs or virtual plant models were proposed as a new-generation crop/plant model that combine both structural and functional approaches to simulate plant growth and development under various environments, and become topical in agroforestry practice [46,52,55,58]. Simultaneously, numerous FSPMs, platforms, and associated tools have been continuously developed worldwide and widely applied to various crops (Table 2), with a particular focus on plant architecture and its interaction with function at the organ level within the context of various environments based on multidisciplinary knowledge [46,53,57]. As a result, FSPMs explicitly allow the feedback between plant architectural performance and underlying physiological activities that can be implemented and verified at different scales (e.g., gene, cell, tissue, organ, plant, population) (Fig. 1), providing a thorough understanding of complex traits across scales [33,53,59–62]. In general, FSPMs can provide more detailed structural and functional attributes of plants compared to typical crop models, and therefore more options can be sought to account for quantitative genetic variation in plant traits. This means that more effort should be paid to parameterize FSPMs. For example, simulations are computationally expensive, and their complexity poses difficulties for the rigorous application of statistical methods for parameter estimation and model evaluation based on experimental measurements [53]. Taking advantage of advanced genomics, phenomics and computational technologies will allow us to answer the question of how to guide functional phenotyping using FSPMs to support breeding in the coming decades [46,55,56].

**Table 2.** The list of FSPMs/modules, a brief description of characteristics and basic functions.

Model	Morphological traits	Functional traits	Plants	References
GreenLab	Weight of each internode, petiole and blade, diameter and length of each internode and petiole, leaf size	Organogenesis and organ expansion, water use efficiency, carbon allocation, phenotypic plasticity resulting from competition among sinks for resources	Rice, wheat, maize, rice, beetroot, sunflower, pine, tomato, cucumber, teak, pepper, chrysanthemum, coffee, maple, poplar, Arabidopsis	[53,65,71,115,163]
ADEL-Maize/Wheat	Leaf lengths and widths, the midrib curvature, distribution of leaf azimuth	Photosynthetically active radiation interception, biomass production and allocation, biomass availability regulate growth	Maize, wheat	[120,164–167]
GRAAL	Leaf (lamina and sheath) length, internode length and diameter, first-order and second-order root length and diameter	Biomass production and allocation, the kinetics of root/shoot ratio, priority between organs, periods of plant sensitivity, and plasticity to carbon availability	Maize	[168]
EcoMeristem	Organ dry weight, leaf area, leaf and tiller number, senescent leaf number, organ length	Development rate, carbon assimilation, organogenesis and competition among growing organs for assimilate, photosynthetically active radiation interception	Rice	[59,78,97]
NEMA	Leaf, sheath, internodes, and chaff	Nitrogen acquisition and distribution, C–N interaction, photosynthetically active radiation interception	Wheat	[96]
FM	Phyllotaxy, upward angle, leaf area, biomass, growth rate	Photosynthesis, respiration, sugar-starch partitioning, carbon allocation, phenology, radiation use efficiency	Arabidopsis	[85,169]
LIGNUM	Tree crown 3D architecture	Photosynthesis, respiration, carbon allocation, growth and development of organ	Scots pine, olive trees	[170,171]
L-PEACH	Apices, stem, leaf, and fruit weight, canopy 3D architecture	Light interception, photosynthesis, carbon accumulation, flow and partition among organs, responses to environment, water extraction	Peach	[66,172]
MAppleT	Branching angle, internode length, top shoot diameter, the total number of shoots and leaf, leaf area, integrated projected leaf area	Light interception, development of apple trees in interaction with gravity	Apple	[113,173,174]
X-Palm	Fruit dry weight, fruit number/bunch, bunch dry weight/number, oil yield	Source/sink ratio regulates inflorescence status and yield components, competition among bunches, competition between fruit size and number	Oil palm	[175]
GrapevineXL	Leaf length/width, petiole/internode length, the declination angle between the petiole and internode, the declination angle between leaf blade and petiole	Radiation absorption, photosynthesis, transpiration, stomatal conductance, leaf water potential, xylem water potential, whole plant root conductance, water transport	Grape	[176,177]
Helios	Canopy 3D architecture	Radiation transport, the surface energy balance, stomatal conductance, canopy photosynthesis, water usage and photosynthesis at leaf-scale variability contribute to whole-plant and -canopy fluxes	Orchard	[178]
RATP	Canopy 3D architecture, leaf orientation/area, crown volume	Radiation absorption, transpiration, canopy photosynthesis	Maize, walnut tree, apple	[107,179–181]
3dCAP	Internode length, leaf length/area/position, sheath length, panicle primary branch number, panicle length, specific leaf weight, grain/tiller number, tiller/leaf angle	Light interception, radiation use efficiency, canopy photosynthesis, canopy vertical nitrogen distribution	Rice, soybean	[32,87,88,182]
Tardieu–Davies model	Leaf elongation rate	Water transport, stomatal conductance, transpiration, circulation of water and ABA in the xylem, leaf and root water potential	Maize, grape	[109,176,183]
SUGAR	Dry weight	Carbon flows between sugar forms, sucrose, sorbitol, glucose, and fructose contents, leaf/fruit ratio	Peach, grape, cucumber, tomato, eggplant, pepper, strawberry, apple, nectarine, clementine, kiwi	[60,184–186]
LEAFC3-N	Leaf senescence	Specific leaf nitrogen, photosynthesis, stomatal conductance, daily carbon fixation	Wheat, oilseed rape, barley	[187]
ROOTMAP	Root diameter, root hairs, root order, root growth rate, branching density and direction	Biomass accumulation, root system plasticity, root proliferation, potential transpiration, local water potential, nutrient acquisition and flow, root exudation	Wheat, weed, lupin, ryegrass, pea, legumes	[188–190]
SimRoot	Root diameter, secondary growth, loss of cortex due to secondary growth, root hairs, root cortical aerenchyma, root growth direction	Resource acquisition and utilization, water-use efficiency, tension difference between soil and xylem, conductivity of the root segment, root exudation, root respiration	Arabidopsis, common bean, maize, squash, lupin, rice	[1,51,191–193]
Root Typ	Elongation rate and duration, tropism, branching density, age, diameter, type of segments, root decay–abscission	Root–soil interaction	Rubber tree, lupin	[194,195]
SPACSYS	Root diameter, root length, root size and distribution, root growth rate	Potential transpiration, water dynamics, C and N cycling among organs, soil and microbes, root death and decay, root exudation	Grasses, wheat, barley, peas, maize, trifolium	[196–198]
R-SWMS	Root age, volume, diameter, root sensitivity to temperature and gravitropism with root order, root radial and axial conductivity evolution with root age, root growth rate, root direction	Root hydraulic architecture, water and nutrient acquisition, transpiration, water flow, solute transport, hormonal transport in the xylem, manipulation of rhizosphere soil properties	White lupin, maize, ryegrass	[67,68,199]
RootBox	Root diameter, root growth direction, root length density, root hairs, mycorrhiza	Root–soil interaction, water and nutrient acquisition, root exudation, root mortality	Maize, pea	[200,201]

Initially, the classical processes in FSPMs are implemented at the organ scale, e.g., leaf photosynthesis [63], respiration [64], carbon allocation [53,65,66], absorption and transportation [67–70], growth and development [62,71,72], and response to environments [1,52,73]. Nowadays, FSPMs can also be extended to the gene or community level [45,46,55,57] (Fig. 1). This allows external agronomic performance to be mirrored to fundamental molecular regulation. For example, when plants grow in clusters at high planting densities, the leaves at the bottom will have shade avoidance syndrome (e.g., tillering ceased earlier, accelerated petiole elongation, increased leaf angle, etc.); however, the consequences of the physiological and molecular regulations of shade avoidance syndrome are difficult to assess [42,73]; interestingly, Pantazopoulou et al. [42] used an FSPM of Arabidopsis constructed with simulation platform GroIMP to study the shade avoidance syndrome from organ responses to plant performance, indicating that the model can help to better understand the complex link between physiological regulation of shade avoidance at the organ level and plant performance in the population.

To date, the fundamental molecular mechanisms in determining many aspects of plant growth and development such as germline development and fertilization [74], leaf shape [20], plant height [75], nonphotochemical quenching [76], etc. are made clear, which provide the foundation to develop metabolic and developmental models in advancing FSPMs [77]. It is shown that the framework of FSPMs allows assisting molecular design breeding by directly linking up model parameters to quantitative trait loci (QTL) allelic or single nucleotide polymorphism (SNP), thereby enabling one to model the effect of genetic manipulation on phenotypes [55,56,78]. This opens up opportunities to evaluate genetic changes in different environments and explore gene–trait–yield scenarios that cannot be implemented experimentally [61]. Based on this approach, many loci governing the genetic variability of traits have been determined, e.g., grain number [79], leaf elongation [80], ear weight [81], height [82], fresh weight [83], early vigor [78], and lycopene accumulation [84].

FSPMs can also integrate new submodules, e.g., gene network regulation, metabolic reactions, and metabolite transport for cross-scale modeling [52]. As a representative example, Chew et al. [85] constructed a cross-scale FSPM by introducing a carbon dynamic module, a photothermal module, and a photoperiodism model. Modeling results reveal the developmental control of leaf production and offer a tool in explaining phenotypic changes caused by overexpression of miR156. Song et al. [86] developed a 3D canopy photosynthesis system (3dCAP) to simulate individual photosynthesis-related metabolic reactions and their key regulatory mechanisms by crossing scales from molecular to canopy level using a C_3_ photosynthetic biochemical model and a 3D canopy structure model, which has been successfully applied to rice [86,87], wheat [88], and soybean [32]. To quantitatively analyze the impacts of leaf anatomy on photosynthesis, Xiao et al. [89] and Retta et al. [90] proposed two 3D CO_2_ reaction–diffusion modules for C3 and C4 plants, respectively, and combined these modules with the corresponding types of photosynthetic biochemical models, highlighting the significance of leaf structural plasticity in improvement of crop photosynthesis.

## FSPMs Supply Rich Functional Targets for Phenotyping

As discussed in the above texts, FSPMs flexibly integrate modules from different scales within a system biology framework, allowing mechanistic modeling multiscale crop performance in dynamic competitive environments. In a scenario modeling, model users enable the retrieval of rich functional targets, particularly inherent traits (Fig. 1). For example, in typical crop modeling, dry matter production is proportional to the intercepted radiation or plant transpiration, and the corresponding proportionality factor is called the radiation use efficiency (RUE) or water use efficiency (WUE), respectively [6,53]. Chang et al. [87], Wu et al. [34], and Zhang et al. [91] showed that biomass production can be replaced by integrating the total photosynthetic rate for all leaves within the canopy with explicitly incorporating biochemical and mechanistic principles, e.g., Farquhar, von Caemmerer, and Berry photosynthesis model (FvCB model) [92], as this approach is able to account for more details of fundamental biology than empirical models [45,46,93]. As such, more functional traits related to leaf photosynthesis can be profiled, including Rubisco carboxylation rate, electron transport rate, photorespiration, dark respiration, stomatal conductance, mesophyll conductance, transpiration, WUE, and specific leaf nitrogen [63,92,94]. This undoubtedly provides specific targets for breeders to improve crop yield via manipulating leaf photosynthetic process under different environments. Similarly, the extinction coefficient is commonly used to describe the efficiency of leaf light interception in traditional crop growth models and FSPMs due to its high computational efficiency, but neglecting the variation in canopy space [95]. In contrast, the approaches that integrate 3D canopy architecture and ray tracing provide more accurate canopy light distribution and interception, thus offering many target traits, such as tiller number, tiller angle, leaf angle, Rubisco carboxylation rate, and electron transport rate [32,87]. To accurately model the photosynthesis rate and optimize canopy carbon gain, Bertheloot et al. [96] developed a nitrogen economy model within plant architecture (NEMA) by linking nitrogen fluxes to nitrogen concentration and physiological processes, which simulates the nitrogen content of each photosynthetic organ, and how the environmental factors regulate their nitrogen content.

Within a plant, the carbon assimilate availability determines the growth of competing organs, which is usually represented by the ratio of synthesized biomass supply to demand in FSPMs [59,78,97]. For example, the concept of “sink regulation” is adopted in the GreenLab model, and the biomass change of an organ is calculated according to the inverse implicit parameters (relative sink strength) [65,71]. Recently, Chen et al. [62] developed a new model for simulating fruit growth by coupling a biophysical model of fruit growth with the kinetics of sugar metabolism. This method would be a more mechanistic approach, although the accuracy of simulation still needs further validation when scaled up to plant or canopy level. Compared with carbon assimilate availability, water availability is more directly related to plant growth under drought stress [98,99]. Dynamic simulation by introducing turgor-driven plant growth into an FSPM provides insights into the variations in sap flow or stem diameter, which can be used as a tool to better understand plant performance under water deficit [100].

Despite the fact that the modeling work in root systems lags far behind that in shoot systems due to the complex soil environment and the lack of tools to capture 3D root architecture [53], there is increasing interest in modeling 3D root systems for FSPMs. Root growth and function are interdependent with shoots in determining the capacity for foraging and acquiring resources in the soil [101]. Dupuy et al. [102] proposed a theoretical framework that describes the dynamics of root density distribution as a function of individual root developmental parameters, such as rates of lateral root initiation, elongation, mortality, and gravitropism. In parallel, Rangarajan and Lynch [1] demonstrated that modeling root system architecture facilitates the prediction of functional attributes including root growth angle (shallow for phosphorus uptake and deep rooting angle for nitrogen capture), lateral root branching density, and length for nitrate uptake. For example, the interaction between root system architecture and the dynamic and spatial patterns of water [68] or nitrogen [69] uptake is modeled, capturing the water/nitrogen transport in the soil and availability to plants, and calculating the adaptive changes in root architecture to this availability. The performance of a plant as a whole results from the interactions between these organs and the integration of the processes throughout the whole plant [45,55,57,59]. Consequently, much remains to be done in modeling root systems and progressively integrating them with shoot systems for intact FSPMs.

In FSPMs, the existing basic modules already allow for identifying many functional traits, and if more are desired, mechanical modules for the relevant processes need to be developed to capture the target functional traits. For example, to link photosynthesis and nitrogen status, Zhu et al. [103] developed a photo-acclimation model to simulate nitrogen partitioning among major photosynthetic proteins, thus enabling prediction of the proportion of absorbed light energy allocated to photochemistry, heat dissipation, and fluorescence emission. Compared to the coarse-grained FvCB model, the fine-grained *e*-photosynthesis model can explicitly simulate the target enzymes and processes for engineering, providing a bridging platform for understanding and modifying targets at the plant scale [103]. Subsequently, Song et al. [86] used a 3D architectural model and ray tracing algorithm to simulate the light distribution within canopy, and further combined this with previous *e*-photosynthesis models to construct a mechanistic canopy photosynthesis model. The scenario analysis revealed that modifying chlorophyll concentration coupled with strategic reinvestment of conserved nitrogen have the potential to support substantial increases in canopy photosynthesis and yield. In a recent continuity study, Mao et al. [104] constructed transgenic rice with amiRNA targeting YGL1, generating different lines with different leaf chlorophyll contents and antenna sizes. They found that reducing antenna size by inhibiting chlorophyll synthesis could improve light distribution and canopy photosynthesis while maintaining photosystem II efficiency. Therefore, users can choose or develop modules according to their needs, and further identifying target functional trait to accelerate the development of superior germplasm resources. Advanced multi-omics and plant phenomics technologies offer an unprecedented capacity for parameterization and validation of model. At the molecular scale, transcriptomes, proteomes, and metabolomes make it possible for parameterizing mechanistic models down to the actual molecular components involved [23,85]. At the leaf or even the whole plant scale, progress in plant phenomics has been achieved in high-throughput monitoring functional traits such as photosynthetic status, water, and pigment content (Table 1).

## Applications of FSPMs in Functional Phenotyping

FSPMs have been shown to be a promising approach in assisting phenotyping functional traits. Paradigms were shown here demonstrating such potentials. Occlusion is an inevitable issue for phenotyping canopy functional activities [105,106], particularly in plants clustered together at high planting densities or at later developmental stages. Functional traits like specific leaf area, nitrogen content, photosynthesis-related traits, and RUE are difficult to measure accurately using conventional high-throughput phenotyping techniques. To overcome this, Cabrera-Bosquet et al. [107] proposed a method to evaluate the light interception and RUE for thousands of plants, coupling the radiation absorption, transpiration, and photosynthesis (RATP) model and 3D virtual plants. They demonstrated that light interception and RUE largely varied with maize lines that differed in leaf angle and area. Similarly, Liu et al. [108] precisely estimated the green area index of wheat by integrating a 3D canopy structure model and terrestrial light detection and ranging (LiDAR), which was then used to derive RUE. Within plants, Rebolledo et al. [78] investigated the genetic architecture of rice early vigor using the EcoMeristem model. The results suggested that metabolic traits (hexose content), leaf size, and leaf appearance rate aid in understanding the complex genetic architecture of early vigor. To simulate the effects of the environment on the variables at varying time resolutions, Tardieu et al. [109] updated the Tardieu–Davies model through combining with equations for water and abscisic acid (ABA) fluxes; it results in a dynamic model able to simulate the coordination of the controls of stomatal aperture, transpiration, leaf growth, and abscisic acid. A greater number of phenotypes can be captured in a simulation than in the field or greenhouse, particularly root phenotypes [110]. Postma et al. [111] used the OpenSimRoot model to realize the simulations of carbon, water, and nutrient acquisition and utilization in maize and wheat. To unravel the dynamics of root water uptake in situ, Koch et al. [67] combined the root-soil water movement and solute transport (R-SWMS) model with 4D tracer observations. They identified that 76% of the transpiration was extracted by third-order roots, which represent 70% of the total root length. The result suggests that detailed tracer experiments combined with FSPMs can help to decipher the mechanisms underlying root water uptake. Thus, this model can help to identify optimal plant properties for breeding crops with greater WUE.

It is necessary to predict crop performance before crop physiologists may take action in breeding programs [52,81] since traits, especially functional types, differ in their effect on crop performance and this effect may vary with environment [77,112]. FSPM is a promising tool by which the consequences resulting from functional traits can be tested in specific environments [52,113]. For example, the effects of future elevated CO_2_ concentrations on plant performance are difficult to determine [52]. Previous studies were based on the Free-air CO_2_ enrichment (FACE) systems, where the high cost of the experimental facility limits the related research progress. Surprisingly, Rakocevic et al. [114] studied the influence of elevated CO_2_ concentrations on structural and functional changes in coffee trees using a CoffePlant3D model, as a result of which the coffee tree compensates for the loss of leaf area through improved photosynthesis of leaves and whole plants (e.g., increased stomatal conductance and leaf photosynthesis in the middle and upper canopy layers, increased WUE). For soybean, Song et al. [32] used a 3D canopy model parameterized based on a soybean FACE system and found that elevated CO_2_ concentrations not only increased LAI at early developmental stages, but also increased the proportion of leaves under Rubisco-limited photosynthesis from 12.2% under low photon flux density to 35.6% under high photon flux density at later developmental stages. This demonstrated the synergetic effect of CO_2_ and light on crop growth under elevated CO_2_ conditions. Elevating CO_2_ concentrations may lead to more frequent high-temperature and drought events. To study plant development under limited soil water availability conditions, Braghiere et al. [44] proposed an integrated model of 3D shoot architecture and biomass growth with a 3D root system, demonstrating that soil water availability has a stronger influence on photosynthesis than the light environment.

FSPMs can also serve as a virtual phenotyping platform, providing the possibility to mine functional genes and plant traits faster that optimize use of resources [56,84]. As an early example, the phenotype of 2 detected QTL genotypes (tillering and grain number) was simulated using a barley FSPM [79]. Subsequently, Letort et al. [81] modeled QTL detection for parameters of the GreenLab model on a virtual mapping population built from a simple genetic model. They defined the ideotype for maximum yield based on the model parameters and the associated allelic combination. Based on this approach, Kang et al. [115] optimized the sink parameters of tomato by combining GreenLab with the particle swarm optimization algorithm, and found that the ideotype tends to have fast leaf and internode expansion, slow fruit expansion, and high fruit sink strength. Luquet et al. [59] showed that the genotypic development rate is a major driver of early vigor in rice under stress-free conditions. Subsequently, Rebolledo et al. [78] found new regions related to early vigor by coupling nonstructural carbohydrates and EcoMeristem model parameters, providing additional information on the genetic control of early vigor. To better understand the interactions between fruit expansive growth and sugar metabolism, Chen et al. [62] developed an integrative model to explore the trade-off between size and sweet of fruit. A virtual experiment predicts that tomatoes can be bigger and sweeter when both biophysical property-related factors (phloem hydraulic conductivity, phloem osmotic pressure caused by the solutes other than sugars, and cell wall extensibility) and transmembrane transport property-related factors (proton-motive force, the capacity of the tonoplastic sucrose carrier, and the capacity of active sugar transport across plasma membrane) are simultaneously manipulated. As a virtual phenotyping platform, FSPMs can help to support the decision cycle of plant performance analysis by integrating different traits into a spatial–temporal whole plant simulation.

To cope with future climate change, new selection criteria have been proposed for various crops. For example, Drewry et al. [116] used a biophysical canopy model (MLCan) to simulate soybean canopy photosynthesis and productivity under future climate change and found that modified soybean canopy attributes (i.e., LAI and its vertical profile, leaf angle distribution, and shortwave radiation reflectivity) can not only increase yield (7%) with no change in water use or albedo, but also increase water use (13%) or albedo (34%) with no loss in productivity. Picheny et al. [113] coupled an FSPM for apple tree (MAppleT) with a multi-objective optimization formulation to obtain the optimal integrated projected leaf area, and the results showed that longer internodes and higher LAI allow building an apple tree ideotype, and proposed 4 optimal tree phenotypic compositions. To better assess the yield consequences of photosynthetic manipulation under water-limited conditions, Wu et al. [34] introduced a coupled photosynthesis–stomatal conductance (A-g_s_) link to a diurnal canopy photosynthesis simulator (DCaPS), enabling the evaluation of photosynthetic manipulations under future climatic conditions when combined with reliable climate predictions. Simulations showed that simultaneous optimization of Rubisco activity, electron transport rate, and mesophyll conductance produced the greatest yield increase in wheat, while the combination of Rubisco activity and electron transport rate was most effective in sorghum in water-limited situations. Optimized root architecture benefits greater nitrogen availability, and Ajmera et al. [51] evaluated the utility of combinations of root architectural traits and different lateral branching densities for plant growth under low nitrogen by coupling OpenSimRoot with a crop model ORYZA_v3. Due to the synergism among root architectural phenotypes, several integrated root phenotypes with higher shoot biomass are identified, and these optimal root phenotypes are predicted to have up to 80% greater yield than the reference cultivar IR64 under low nitrogen. Overall, FSPMs can delicately decipher the influence of changes in functional or structural traits on crop performance, thus providing valuable traits contributing to an ideotype that is best adapted to targeted environmental scenarios in phenotyping.

## Challenges in FSPMs + Plant Phenomics

FSPMs combine the performance of 3D plant and canopy structure over time with specific physiological behavior [55] and have become important tools for studying relationships between structure and function [117]. While there is an increasing interest in using FSPMs to compare plants derived from different genetic sources, accurately modeling 3D architecture is paramount as this significantly affects the light distribution and interception, and the subsequent physiological processes such as canopy photosynthesis and transpiration [113,117]. However, constructing and assessing the 3D structural of virtual plants is still methodologically complex [118]. The rise of plant phenomics constitutes a major evolution, in changing the data availability of both plant structure and function in breeding, and poses an unprecedented challenge in development of FSPMs [14,46]. For example, 3D reconstruction methods, such as LiDAR [118], multiview stereo systems [33], and time-of-flight systems [18], open up new prospects for phenotyping plant structure as it allows quick and effective in situ collection of 3D information at the plant scale. Using the LiDAR method, Perez et al. [118] assessed the capacity of a 3D architectural model, compared light interception among oil palm progenies, and revealed another practical use of LiDAR for evaluating the radiative environment of plants. Although existing advanced phenotyping tools are capable of obtaining visually realistic 3D plant morphology and extracting the data required for structural model building (Table 1), extracting accurate information on structural and functional traits remains a challenging task when faced with branch occlusion [119]. In contrast, FSPMs can offer rich functional traits (Table 2; details are shown in the “FSPMs Supply Rich Functional Targets for Phenotyping” section). To some extent, therefore, FSPMs and plant phenomics are complementary.

**Fig. 2.**
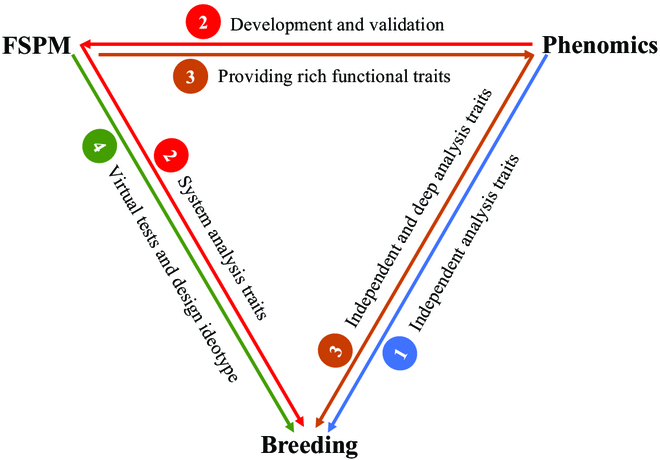
Schematic diagram of a trans-disciplinary integrated approach in breeding systems, highlighting the integration role of FSPMs in understanding G × E × M interactions. (1) High-throughput phenomics platform enables the researchers to profile structural and functional features of shoot and root with hundreds of genotypes; such traits can be directly used for genetic analysis. (2, 3) The high-throughput phenomics platform accelerates the development and validation of FSPMs, making them more accurate and useful for predicting plant growth and development; in turn, FSPMs provide more functional traits and strategically guide the deployment of phenomics in a specific breeding program. Further, FSPMs can systematically analyze and predict the given traits in virtual scenarios. (4) According to the idea and demand of breeding, FSPMs allow breeders to develop virtual tests and design an ideotype that is best adapted to the targeted environment.

Plant phenomics is focused on the understanding of variations in plant phenotypes resulting from genotypes by environments, but the physiological processes or traits are behind the exploration of morphological trait phenotyping. Therefore, to establish robust physiological traits, new technologies must break through the bottleneck and achieve high-precision, cross-scale, ultrasensitive, and high-throughput physiological process observation, especially at the microcosmic level, which will open up new opportunities to explore the complex interactions between genotypes and environments. As previously discussed, the paradigm of FSPMs allows offering more functional traits for guidance of phenomics, virtual tests, and breeding design. To achieve optimal combination by virtue of FSPMs, undoubtedly, FSPMs still need to evolve in several ways to embrace the advancement in molecular biology, molecular design breeding, and phenomics techniques (Fig. 2).

Given the complexity of genotype × environment × management in breeding, it can be difficult to determine how much a specific genetic modification contributes to the effect on the whole plant. Under various agroclimatic conditions, modification of functions or pathways does not lead to a proportional increase in final yield [30,56]. For instance, no change in wheat grain yield was caused while the maximum carboxylation rate of Rubisco was enhanced by 20% [34]. There is an increasing interest to integrate models at multi scales of spanning from gene networks to plant and population in the development of FSPMs [52,55]. Chew et al. [85] constructed a multiscale FSPM for Arabidopsis that integrated gene dynamics, carbon allocation, and organ growth and development responses to internal and external signals. Ultimately, such approaches using coupled multiscale models provide a quantitative explanation for the gene regulation under defined environmental scenarios.

To develop and apply FSPMs in breeding and genetic engineering, the modelers need to construct more mechanistic and credible modules representing the biological process at the multiscales, thereby introducing more independent and relevant biological parameters with genetic diversity into models. FSPMs have been achieved in simulating crop photosynthesis from the molecular to the population scale [88]. However, a key module for modeling crop biomass allocation to individual organs in FSPMs is still largely dependent on empirically mathematical fitting, e.g., GreenLab [65], ADEL-wheat [120], or EcoMeristem [97]. Within a plant, the assimilates are carried from source to sink driven by a turgor pressure difference through the osmotic effect of sucrose [121]. As such, sink strength can be considered as both fundamental cellular activities driven by sucrose unloading, and enzymatically degraded into hexoses that power and support the growth of sinks, which is regulated through a variety of enzymes and genes [122]. However, the existing carbon allocation and starch turnover models are empirically parameterized only locally and limited in their expansions [85]. Utilizing the regulatory mechanism of starch turnover by the clock and sugar sensing may enable one to predict the allocation and use of photosynthate in various environmental conditions [52]. Chen et al. [62] studied the genotype × environment × management on fruit growth and carbon metabolism by introducing an enzyme-based kinetic model of sugar metabolism to an FSPM. Therefore, the development of new modules, or updating old modules, or integrating different modules will help to expand research coverage and capture more critical interactions within a dynamic biological system from the genome to the field.

Unlike vegetative tissues, i.e., roots, stems, and leaves, reproductive organs are seldom modeled despite their critical roles in crop production, particularly under abiotic stress. Take maize as an example, silks must rapidly elongate during the flowering stage to emerge from bracts and then receive pollen grains originating from the apical male tassels [123]. In this process, reproductive organs are particularly sensitive to abiotic stresses compared with vegetative organs; for example, water deficit reduces silk elongation rate and further increases anthesis-silking interval [123,124]; in addition, heat stress limits the release and viability of pollen grains [125], resulting in failure of fertilization. To predict the phenotypes of reproductive failure and yield loss under stress, the dynamic modeling of reproductive development and processes, i.e., silk initiation, silk elongation, pollination, and kernel growth, is urgently needed. Reassuringly, there have been some tentative efforts. For example, combined with a confocal microscope, Richardson et al. [126] developed a model to simulate primordium extension; Shi et al. [127] visually observed pollen germination and fertilization processes; Turc et al. [123] measured the silk elongation rate with a rotational displacement transducer; and Ma [128] coupled source- and sink-limited allocation approaches to simulate kernel-filling processes with a GreenLab-Maize model.

Due to the underground location of root systems, it is challenging to investigate their growth and development in a noninvasive and high-throughput manner in field conditions [1,67]. Despite notable advances in modeling 3D root architectural development over the past decades (Table 1), the phenotyping of many root traits lags due to the lack of high-throughput quantitative measurement below the ground. Root growth and development are the result of intricate interactions between soil, water, nutrient and carbon availability, nutrient perception, and tropisms; they are not or only partially included in root FSPMs [101,111]. In such models, most plant physiological processes are simplified to a set of response curves [129]. While these approaches have been beneficial, they have not facilitated a better understanding of the underlying complexity owing to the absence of biological functionality in the model. Introducing mechanistic approaches into modeling is essential. The future development of root FSPMs should progress toward regulatory networks that more closely imitate plant physiology, possibly even integrating gene-based approaches, which will enable better representation and prediction of the phenotypic plasticity of root systems in a heterogeneous soil environment.

## Conclusion and Prospects

Functional trait phenotyping is a vital mission in the development of plant phenomics for breeding crops. In particular, the functional traits for high-yielding and resistant crops are quantitative and controlled by many candidate genes. The employment of FSPMs allows identifying crop secondary traits by conforming to fundamental principles in systems biology. FSPMs explicitly simulate the complex yield formation and adaptation to non-optimal conditions through embracing the feedback between plant 3D architecture and physiological function from the molecular to whole plant level. FSPMs provide an analytical framework for identifying both functional and morphological traits, which thus offer rich functional targets for plant phenotyping in accelerating plant breeding. Importantly, FSPMs can be used to test hypotheses and scenarios under various environmental conditions, which allow speeding up the selection of ideal traits. Simultaneously, advances in plant phenomics have enabled the rapid reconstruction of 3D canopy architecture and quantification of organ growth dynamics, which also provides an unprecedented opportunity to develop, calibrate, and validate FSPMs. This will result in accelerated co-evolution of FSPMs and plant phenomics, and thus the development of new crop varieties with improved yield, quality, and resilience to environmental stress. Notably, there is still much more work to be done in different disciplines in fulfilling their potential. New technologies must be developed to break through the bottleneck in achieving high-precision, multidimension, cross-scale, ultrasensitive, and high-throughput physiological process observation, especially at the microcosmic level.
